# A *Yarrowia lipolytica* Strain Engineered for Pyomelanin Production

**DOI:** 10.3390/microorganisms9040838

**Published:** 2021-04-14

**Authors:** Macarena Larroude, Djamila Onésime, Olivier Rué, Jean-Marc Nicaud, Tristan Rossignol

**Affiliations:** 1 AgroParisTech, Micalis Institute, Université Paris-Saclay, INRAE, 78350 Jouy-en-Josas, France; macarenalarroude@gmail.com (M.L.); djamila.onesime@inrae.fr (D.O.); jean-marc.nicaud@inrae.fr (J.-M.N.); 2 INRAE, MaIAGE, Université Paris-Saclay, 78350 Jouy-en-Josas, France; olivier.rue@inrae.fr; 3 INRAE, BioinfOmics, MIGALE Bioinformatics Facility, Université Paris-Saclay, 78350 Jouy-en-Josas, France

**Keywords:** *Yarrowia lipolytica*, pyomelanin, chassis strain, aromatic amino acids

## Abstract

The yeast *Yarrowia lipolytica* naturally produces pyomelanin. This pigment accumulates in the extracellular environment following the autoxidation and polymerization of homogentisic acid, a metabolite derived from aromatic amino acids. In this study, we used a chassis strain optimized to produce aromatic amino acids for the de novo overproduction of pyomelanin. The gene *4HPPD*, which encodes an enzyme involved in homogentisic acid synthesis (4-hydroxyphenylpyruvic acid dioxygenase), was characterized and overexpressed in the chassis strain with up to three copies, leading to pyomelanin yields of 4.5 g/L. Homogentisic acid is derived from tyrosine. When engineered strains were grown in a phenylalanine-supplemented medium, pyomelanin production increased, revealing that the yeast could convert phenylalanine to tyrosine, or that the homogentisic acid pathway is strongly induced by phenylalanine.

## 1. Introduction

Melanins are a heterogeneous group of polymeric pigments that are widely found in nature, in organisms ranging from microbes to humans. They result from the oxidation and polymerization of phenolic or indolic compounds. The resulting macromolecules have high molecular weights, are negatively charged and hydrophobic, and can vary in color. Based on their biosynthetic pathway of origin, they form three groups: eumelanins, pheomelanins, and allomelanins. Eumelanins are produced when tyrosine and/or phenylalanine is oxidized, generating *o*-dihydroxyphenylalanine (DOPA) as an intermediate compound and, ultimately, a black or brown pigment. Pheomelanins are synthesized in a similar manner, but the DOPA undergoes cysteinylation, resulting in a red-yellow pigment. The allomelanins are a larger group and result from the oxidation of different compounds. For example, catechols, 4-hydroxyphenylacetic acid, tetrahydroxynaphthalene, and homogentisic acid are precursors of different allomelanins. Pyomelanin in particular is a brown pigment that is produced via the oxidation of homogentisic acid (HGA) synthesized from tyrosine [1].

Melanins have diverse applications due to their chemical compositions and physicochemical properties. These pigments protect cells from various environmental stressors: they absorb ultraviolet (UV) light, X-rays, and γ-rays; scavenge reactive oxygen species (ROSs) and free radicals; and serve as ion exchangers [2]. Such properties are beneficial in a wide range of contexts. Because they provide UV protection and have antioxidant effects, they are often incorporated into pharmaceutical and cosmetics products [3,4]; they are also employed in contact lenses and sunglasses [5]. Thanks to their redox behavior, melanins can be used as amorphous organic semiconductors in electronics [6,7]. Moreover, melanins are employed in bioremediation efforts at contaminated sites due to their capacity to act as metal chelators [8]. Finally, melanin can help synthesize silver or gold nanostructures and nanoparticles, which have applications in the food and health industries [9,10].

Melanins can be produced in an ecofriendly way and at a low cost using microorganisms [11]. Several recombinant microorganisms have been constructed to date; the highest melanin yields (28.8 g/L) have been obtained using *Streptomyces kathirae* [12].

The yeast *Yarrowia lipolytica* is known for its natural ability to generate melanins from tyrosine [13], which was discovered when researchers noticed brown pigments appearing on ripening cheeses [14]. It was later understood that the underlying mechanism was tyrosine degradation. The pigment is produced via a biphasic process in which a precursor, HGA, first accumulates in the extracellular environment and is then autoxidized and polymerized, leading to the formation of pyomelanin [13,15]. In addition, researchers have identified the culture conditions under which pigment production is favored—high aeration levels and a neutral pH [13]. Recently, Ben Tahar et al. [16] observed pyomelanin production in the *Y. lipolytica* W29 wild-type strain. In tyrosine-supplemented media, they obtained a yield of 0.5 g/L after 5 days of culture; they also demonstrated the pigment’s antioxidant capacity and low cytotoxicity toward human keratinocytes, meaning the compound could be used as an anti-UV agent in sunscreen. Ito et al. [17] showed that melanin was constitutively synthesized in *Y. lipolytica*, where it helps to sequester metal ions, increasing the species’ tolerance to copper. Apte et al. [9,18] enhanced melanin production in *Y. lipolytica* by culturing the yeast in the presence of l-DOPA. Thanks to its ability to act as an electron exchanger, the resulting melanin was used to synthesize silver and gold nanoparticles. Silver nanoparticles are useful paint additives because of their antifungal properties. Despite this body of work, little is actually known about how *Y. lipolytica* generates melanin. We need a better mechanistic understanding of the production process in this yeast if we wish to engineer higher yields.

**Figure 1:**
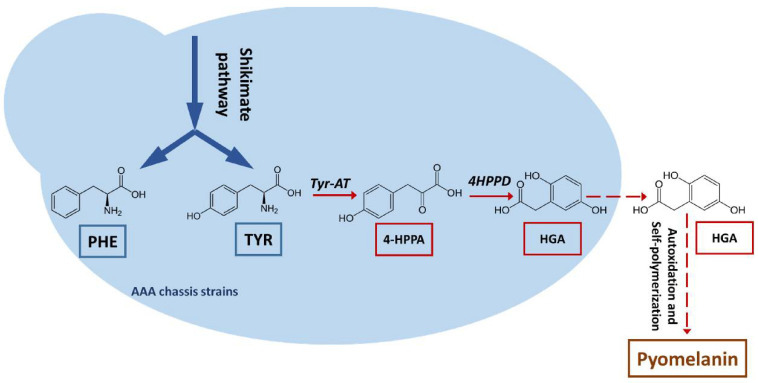
Schematic representation of the pyomelanin biosynthesis pathway. PHE: phenylalanine; TYR: tyrosine; *Tyr-AT*: tyrosine aminotransferase; 4-HPPA: 4-hydroxyphenylpyruvic acid, *4HPPD*: 4-hydroxyphenylpyruvic acid dioxygenase; HGA: homogentisic acid.

In a previous study, chassis strains overproducing tyrosine and phenylalanine were developed by deregulating and overexpressing the shikimate pathway [19]. Such strains are a good starting point for de novo pyomelanin production, circumventing the need for aromatic amino acid (AAA) feeding (Figure 1). In this study, a gene encoding an enzyme involved in pyomelanin production, *4HPPD*, was characterized in *Y. lipolytica*. A “hyperproductive” strain was developed using an AAA-overproducing chassis strain as a starting point. Minimum yields of 4.5 g/L were reached.

## 2. Materials and Methods

### 2.1. Strain and Plasmid Construction

The *Escherichia coli* DH5α strain was used for plasmid propagation. The plasmids constructed in this study were assembled using a Golden Gate (GG) protocol and the building blocks described in Larroude et al. [20]. All expression vectors were assembled using the strong constitutive pTEF promoter and the tLIP2 terminator for each transcription unit [20]. For *4HPPD* overexpression, the YALI0B21846g open reading frame (ORF) was PCR amplified from the W29 strain using the oligonucleotides described in Supplemental Table S1 and cloned using the GG cloning procedure described in [20]. Overexpression of TKL1 was carried out using a JMP62-type plasmid with LEU2ex as a marker and pTEF as a promoter (JME3286) [21]. A vector expressing the CRE recombinase, JME547, was used for marker recovery [22] as needed.

CRISPR/Cas9 vectors for *Yl4HPPD* disruption were constructed using Leu2–Cas9 JME4390 backbone vectors and the oligonucleotides 4HPPD_sgRNA1_Fw and 4HPPD_sgRNA1_Rv (see Supplemental Table S1) using the sgRNA sequence cloning procedure [23].

*Y. lipolytica* was transformed utilizing the lithium acetate method [24]. Mutants were selected on the appropriate media. For CRISPR/Cas9, transformation was followed by outgrowth (as per [23]).

All the *Y. lipolytica* strains used in this study were derived from the Po1d strain (Table 1).

All restriction enzymes were purchased from New England Biolabs. PCR amplification was performed using GoTaq DNA polymerases from Promega (Charbonnieres-les-Bains, France). PCR fragments were purified with a NucleoSpin^®^ Gel and PCR Clean-up Kit from Macherey-Nagel (Duren, Germany), and plasmids were isolated from *E. coli* with the NucleoSpin^®^ Plasmid EasyPure Kit from Macherey-Nagel (Duren, Germany).

### 2.2. Strain Sequencing and Integration Site Identification

The complete genomes of JMY8032 and JMY8208 strains were fully sequenced by Eurofins Genomics (Ebersberg, Germany) using the Illumina Novaseq 6000 platform (paired-end 2 × 150 bp). For DNA extraction, cells were grown at 28 °C in 10 mL of YPD medium for 24 h and harvested by centrifugation. Total genomic DNA was then extracted from cell pellets using the MasterPureTM yeast DNA purification KIT from Epicentre (Madison, WI, USA) according to the manufacturer’s instructions. The resulting DNA pellets were dissolved in 100 μL TE (Tris-HCl 10 mM, EDTA 1 mM, pH 8.0) with 1 μL RNase A (10 mg/mL), incubated at 37 °C for 15 min, and ethanol precipitated. Finally, the DNA was dissolved in Tris buffer (10 mM, pH 8). Raw sequencing data were deposited to the SRA database and are available under the Project accession number PRJNA715500 (data accession number SRR14075915 for strain Y8032 and SRR13999501 for strain Y8208).

Reads were cleaned with fastp v. 0.19.4 [26] with the following options (--cut_mean_quality 30 --cut_window_size 4 --n_base_limit 0 --length_required 100 --low_complexity_filter --correction –cut_by_quality3). Then, overlapping paired-end reads were merged with PEAR [27] with the following parameters (--max-assembly-length 290 --min-assembly-length 20 --min-overlap 20 --*p*-value 0.01). Assembled and unassembled reads were mapped with bwa mem algorithm [28] to the W29 strain reference genome and the cassette sequences. To find the insertion zones, alignments were manipulated with samtools [29] and bedtools [30] in order to keep only pairs whose mates were mapped at the extremities of the cassettes and somewhere on the reference genome, except to the URA3 gene. Finally, remaining reads were visualized with IGV [31] for a manual expertise of region of interest. A total of 3 regions with the insertion were identified in strain Y8208 that were not present in the mother strain Y8032 (CP017556.1:3252866-3252937; CP017556.1:3616128-3616558; CP017557.1:1868257-1868296).

### 2.3. Media and Culture Conditions

The *E. coli* strains were grown at 37 °C in Luria–Bertani medium (10 g/L tryptone, 5 g/L yeast extract, and 10 g/L NaCl) containing either 100 μg/L ampicillin or 50 μg/L kanamycin for plasmid selection.

The *Y. lipolytica* strains were grown at 28 °C in minimal YNB medium containing 10 g/L glucose, 1.7 g/L yeast nitrogen base, 5 g/L NH_4_Cl, and 50 mM phosphate buffer (pH 6.8) or in rich YPD medium containing 10 g/L glucose, 10 g/L peptone, and 10 g/L yeast extract. Uracil (100 mg/L) and leucine (700 mg/L) were added to meet the requirements of auxotrophic strains; hygromycin B (250 mg/L) or nourseothricin (400 mg/L) were added to select for strains. For the AAA bioconversion experiments, tyrosine (1 g/L) and phenylalanine (2 and 7 g/L) were employed as supplements.

The *Y. lipolytica* strains were precultured overnight in 5 mL of YPD medium (28 °C, 180× rpm). The precultures were then centrifuged, washed twice with sterile distilled water, and used to inoculate 15 mL of YNB medium in 100 mL flasks (OD_600_ of 0.05). Cells were grown at 28 °C under agitation (180 rpm) for up to 25 days. Every 2–3 days, the cultures were visually assessed to monitor the appearance of brown pigment. Samples were centrifuged, and the supernatants were used for HPLC analysis or pyomelanin extraction. For each strain, two replicates were used.

Solid media for *E. coli* and *Y. lipolytica* were prepared by adding 15 g/L agar to liquid media.

### 2.4. HPLC Analysis

The supernatants used in the HPLC analysis were treated with 1% (*v*/*v*) trifluoroacetic acid at 4 °C for at least 1 h; they were then centrifuged and filtered (0.22 µm filters) prior to analysis.

An UltiMate 3000 UHPLC apparatus (Thermo Fisher Scientific) was equipped with an Agilent ZORBAX Eclipse Plus C18 Column (4.6 × 100, 3.5 microns) operating at 40 °C. A gradient of acetonitrile and 20 mM KH_2_PO_4_ (pH 2) with 1% acetonitrile was used as eluent. The flow rate was 0.8 mL/min; the percentage of acetonitrile increased from 0 to 10% in the first 6 min and then from 10% to 40% from 7 min to 23 min. From 23 min to 27 min, the flow contained 99% KH_2_PO_4_.

Detection was performed using a diode array detector; measurements occurred at 200, 214, 280, and 310 nm. We looked for the following compounds of interest: Ehrlich metabolites—2-(4-hydroxyphenyl)ethanol (4OH2PE), 4-hydroxyphenylacetic acid (4OHPAA), phenylethanol (2PE), and phenylacetic acid (PAA); AAAs (phenylalanine, tyrosine, and tryptophan); and HGA. Calibration curves were established for each compound using commercial standards from Sigma (St. Quentin Fallavier, France).

### 2.5. Pyomelanin Purification

Cultures were centrifuged (10,000× *g* for 15 min), and melanin was purified from the supernatant via acidification (1 N HCl was added until a pH ~2 was reached), which provoked pyomelanin precipitation. After 24 h at room temperature, the resulting samples were centrifuged (10,000× *g* for 10 min). The brown pellet obtained [32] was then washed, lyophilized, and weighed.

## 3. Results

### 3.1. Production of a Brown Pigment by Yarrowia Lipolytica

Previous research has shown that *Y. lipolytica* can produce a brown pigment, found to be a polymer composed of a core of tyrosine-derived aromatic residues [13]. The pigment was later identified as pyomelanin [16], a compound that results from the autoxidation and polymerization of homogentisic acid (HGA) that has accumulated in the extracellular environment [33].

**Figure 2:**
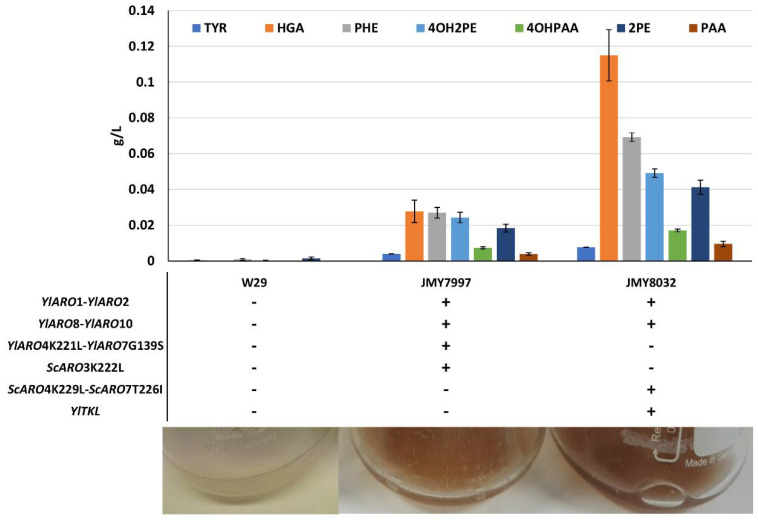
Production of pyomelanin precursors. **Top**: Concentrations of tyrosine (TYR), phenylalanine (PHE), homogentisic acid (HGA), phenylethanol (2PE), phenylacetic acid (PAA), 2-(4-hydroxyphenyl)ethanol (4OH2PE), and 4-hydroxyphenylacetic acid (4OHPAA) in the supernatants of the three strains at 5 days of culture. Values correspond to average of 2 replicates. **Middle**: Strain genotypes. **Bottom**: Images illustrating pigmentation levels in the cultures at 25 days of growth.

To significantly improve de novo pyomelanin production in *Y. lipolytica*, we started off using chassis strains optimized for AAA production. Their shikimate pathway has been strongly engineered—key genes are overexpressed, including unregulated variants of *ARO4* and *ARO7*, whose products play key regulatory roles in the pathway [19]. Such strains have higher tyrosine and phenylalanine content. For these reasons, they served as a good starting point for improving de novo pyomelanin production. First, we compared the abilities of two chassis strains, JMY7997 and JMY8032, and a wild-type strain to produce brown pigment. The genetic modifications made to JMY7997 and JMY8032 are described in Figure 2 and Table 1. After 25 days of culture in minimal YNB medium, the two chassis strains yielded a brown-tinted supernatant, while the wild-type strain did not (Figure 2). Precursor abundance was greatest at 5 days of culture (Figure 2) and tended to decrease over time. This pattern was particularly noticeable for HGA, the direct precursor of pyomelanin.

Pigmentation levels varied across the strains and were clearly linked to genotype—they were higher for JMY8032 than for JMY7997. As mentioned above, the wild type did not show any signs of having produced brown pigment. The concentrations of key precursors—phenylalanine, tyrosine, HGA, and Ehrlich metabolites (derived from phenylalanine and tyrosine)—were quantified for the three strains after 5 and 25 days of culture. We found that all the precursors (and especially HGA) tended to disappear over longer periods of time. At 5 days of culture, precursor levels were much higher in both chassis strains than in the wild-type strain (Figure 2). Each metabolite was at least twice as abundant in JMY8032 compared to JMY7997, a pattern that was particularly pronounced for HGA. These results fit with our visual assessments of pigment levels. Consequently, we used JMY8032 in the subsequent experiments, in which the brown pigment was characterized and its production was boosted.

### 3.2. Characterization of the Brown Pigment

The next phase of the study was to examine the impact of phenylalanine and tyrosine on the growth of the strain JMY8032. The objective was twofold: to confirm that the brown pigment produced by the strain was indeed pyomelanin and to further decipher the pigment’s biosynthesis pathway.

**Figure 3:**
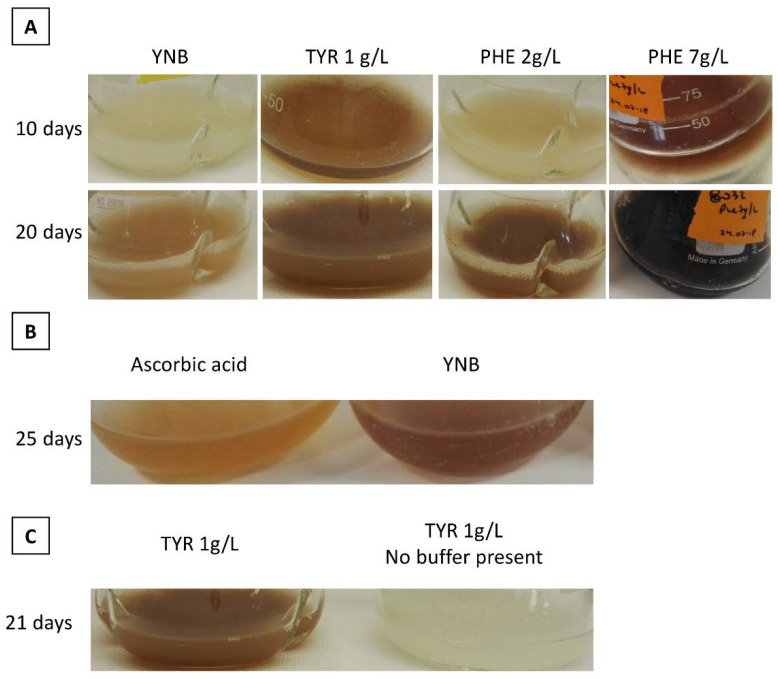
(**A**) Changes in pigment levels over time and in response to tyrosine (TYR) or phenylalanine (PHE) supplementation. (**B**) Inhibitory effect of ascorbic acid on pigment production. (**C**) Inhibitory effect of low pH on pigment production, due to the absence of buffer in the medium. All cultures were performed using the strain JMY8032.

When phenylalanine or tyrosine was added to the culture medium, the brown pigment appeared more quickly. Tyrosine triggered greater pigment production than did phenylalanine (Figure 3A: tyrosine = 1 g/L and phenylalanine = 2 g/L), and pigment levels were highly pronounced after only 10 days of culture. The concentration of tyrosine could not be further increased due to the compound’s low solubility. However, when concentrations of phenylalanine were increased, pigment levels were boosted and time to pigment appearance decreased (Figure 3: phenylalanine = 2 g/L and phenylalanine = 7 g/L). At a phenylalanine concentration of 7 g/L, pigment levels were prominent at 10 days of culture; at a phenylalanine concentration of 2 g/L, 20 days of growth were required to achieve equivalent results. For the wild-type strain, the brown pigment only began to appear after 30 days of culture in YNB medium supplemented with tyrosine (1 g/L). These results differ from those of [16], who observed that a *Y. lipolytica* wild-type strain produced pyomelanin after fewer than 5 days of culture in a medium with the same tyrosine concentration (1 g/L). Here, *Y. lipolytica* wild-type strains did not produce brown pigment under any other experimental conditions. Our findings suggest that the pigment was generated by phenylalanine and tyrosine catabolism.

These results—the marked, rapid appearance of brown pigment (Figure 3A)—concur with those of previous research [13]. Similarly, pigment precursors were found to accumulate in the extracellular environment during the exponential growth phase, and pigment formation occurred during the stationary phase because of precursor oxidation [13].

Then, we assessed whether ascorbic acid had an effect on the brown pigment: this acid acts as an antioxidant and thus inhibits melanin synthesis [34,35]. The addition of 10 mM of ascorbic acid to the media clearly reduced the production of brown pigment, regardless of AAA supplementation (Figure 3B). This result suggests that the oxidation of intermediate compounds was inhibited. In addition, when the medium contained no buffer, no brown pigment was produced, even in the presence of AAA supplementation (Figure 3C). This outcome may have resulted from the acidification of the medium during *Y. lipolytica* growth, as the yeast naturally secretes several organic acids, including citric acid, α-ketoglutaric acid, and pyruvic acid [36], while low pH can impair the oxidation of intermediate compounds.

Another characteristic of melanins is that they are soluble in alkaline solutions but not in water, organic solvents, or acid solutions [37]. We thus evaluated the solubility of the brown pellet obtained after acidifying the supernatant. We found that the pellet was soluble in NaOH but not in water (Supplementary Figure S1), a result that is in accordance with the physicochemical properties of pyomelanin.

### 3.3. Characterization and Improvement of the Pyomelanin Production Pathway

**Figure 4:**
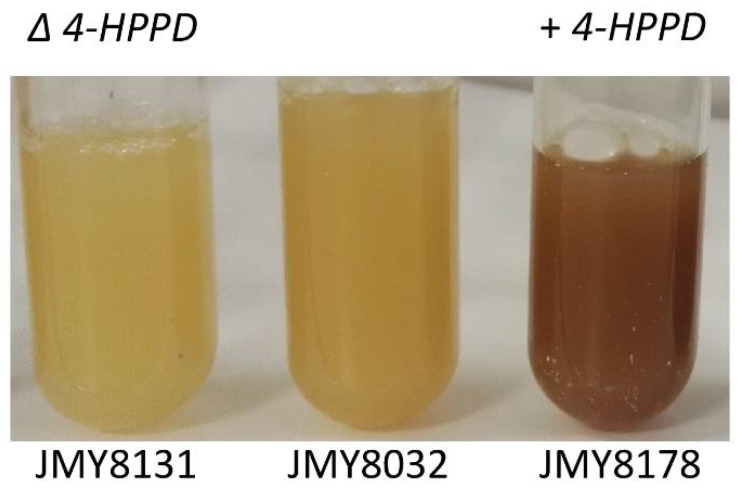
Effect of *4HPPD* disruption and overexpression on brown pigment production in the strain JMY8032 in YPD medium after 4 days of culture.

As previously noted, HGA is the direct precursor to pyomelanin (Figure 1). We thus focused on a gene that potentially encoded an enzyme involved in HGA synthesis, based on prior annotation of the *Y. lipolytica* genome (YALI0B21846g; 4-hydroxyphenylpyruvate dioxygenase, *4HPPD*). We generated strains (background genotype: JMY8032) in which this gene was either disrupted or overexpressed to confirm that the gene was indeed involved in HGA synthesis and that the pigment produced by JMY8032 was derived from HGA. Gene disruption was carried out using CRISPR-Cas9; the resulting strain, JMY8131, had a 16-nucleotide deletion at the PAM site (verified by sequencing; data not shown). As expected, the strain was unable to produce brown pigment in any of the media tested (Figure 4). In contrast, the strain in which the gene was overexpressed, JMY8178, displayed increased levels of pigment formation. A brown tint was visible after 12 days of culture in YNB medium and after just 3–4 days in YPD medium (Figure 4).

Among the clones overexpressing the *4HPPD* cassette, one appeared to become much browner much more quickly than the others. To determine the cause, the clone (JMY8208) and its parent strain (JMY8032) were fully sequenced. The results showed that all the expected cassettes were present (i.e., those involved in the overexpression of the AAA pathway). This “hyperproductive” strain (JMY8208) appeared to have a higher number of *4HPPD* cassettes. Sequencing revealed that the cassettes had been inserted at three different loci in the *Y. lipolytica* genome, meaning that the strain had three copies of the *4HPPD* overexpression cassette (See Materials and Methods). The three cassette copies occurred in intergenic regions, so the high level of pyomelanin production was probably not related to the characteristics of the integration sites. This three-copy strain thus shows great promise for increasing pyomelanin yield.

**Figure 5:**
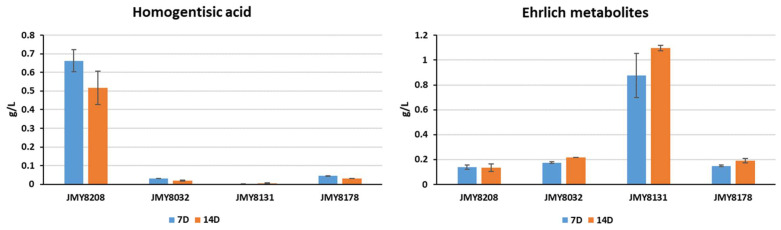
Production of homogentisic acid and Ehrlich metabolites (in g/L) by the four study strains (JMY8032, JMY8131, JMY8178, and JMY8208) in YNB medium. The individual quantities of 2-4OHPE, 4OHPAA, 2PE, and PAA were summed to obtain the total quantity of Ehrlich metabolites. 7D: 7 days of culture; 14D: 14 days of culture.

All four strains—JMY8032, JMY8131, JMY8178, and JMY8208—were grown for 2 weeks in YNB medium to evaluate their levels of precursor compounds, notably those of HGA and the Ehrlich metabolites. As expected, the *4HPPD*-deletion strain (JMY8131) generated no HGA; it also produced a much larger quantities of Ehrlich metabolites than did the parental strain (JMY8032) (Figure 5). Compared to the parental strain (JMY8032), the strains with one and three copies of *4HPPD* (JMY8178 and JMY8208, respectively) produced slightly lower quantities of Ehrlich metabolites and much larger quantities of HGA. Furthermore, the three-copy strain produced quantities of HGA that were up to 15 times higher than those of the other strains (Figure 5). These findings explain the three-copy strain’s “hyperproduction” of the pigment.

It was not possible to perform an HPLC analysis of the precursors occurring in the YPD medium. However, because the three-copy strain produced pyomelanin much faster in this medium (Supplemental Figure S2), we were able to evaluate its pyomelanin yield after just 5 days.

After 5 days of culture, we took a 20 mL sample of the three-copy strain (JMY8208) grown in YPD. Pyomelanin was precipitated from the resulting supernatant. After six rounds of precipitation with 35% HCl, no more of the pellet was recovered even if the supernatant was still very brown. The portion of the pellet that had been recovered was lyophilized and weighed, yielding 0.0891 g of pyomelanin precipitate (4.5 g/L). This value was clearly an underestimate of pigment production, given how brown the supernatant remained. By comparison, the pigment yield for the parental strain (JMY8032) after 40 days of culture was just 1 g/L.

## 4. Discussion

This study examined the role played by the *4HPPD* gene in pyomelanin biosynthesis in *Y. lipolytica*. We managed to create a strain with higher, faster pyomelanin production that was engineered from a chassis strain optimized for AAA production and that expressed multiple copies of *4HPPD*. Indeed, its pyomelanin yield is the highest to date to be obtained in this yeast without AAA feeding. The three-copy strain thus holds promise for several applications, including synthesizing nanostructures in a more sustainable way [16]. 

Our findings indicate that the enzyme encoded by *4HPPD* plays an important functional role that must be considered when using *Y. lipolytica* to produce aromatic compounds. The AAA-overproducing chassis strain already generated a large quantity of pyomelanin, underscoring that AAAs are leaking through the pyomelanin pathway, as evidenced by the rapid accumulation of HGA. Therefore, when *Y. lipolytica* is used to generate aromatic compounds like naringenin, resveratrol, or Ehrlich metabolites, this pathway should be deleted. Indeed, this is exemplified in the strain JMY8131 where the *4HPPD* gene was deleted, the amount of Ehrlich metabolites increases by a factor of 5 compared to the parent strain.

Past research has indicated that pyomelanin is mainly derived from tyrosine [13,33,38]. However, we discovered that brown pigment was also produced when the growth medium was supplemented with phenylalanine. Phenylalanine can be irreversibly converted into tyrosine via phenylalanine 4-monooxygenase in a variety of organisms, including bacteria [39]. Even though this process has not yet been described in yeasts and BLASTP analysis did not reveal any orthologs, we found evidence that this biosynthetic pathway might be present in *Y. lipolytica*. Given that the brown pigment appeared more slowly when the medium was supplemented with phenylalanine than with tyrosine, it could be that, in the former case, pigment production could only occur after the phenylalanine was converted to tyrosine. Another hypothesis is that phenylalanine supplementation may have a regulatory effect on the conversion of tyrosine to HGA. As a result, tyrosine degradation may accelerate, HGA levels may climb, and pyomelanin may accumulate. However, the very dark tint obtained with phenylalanine supplementation suggests that it comes from phenylalanine conversion as de novo synthesis of tyrosine in this strain could hardly promote such a level of pyomelanin production.

## Figures and Tables

**Table 1:** YlARO4K221L and YlARO7G139S are both the mutated forms of *Y. lipolytica* Aro4 and Aro7, corresponding to the feedback-inhibited counterpart of the *S. cerevisiae* enzymes; ScARO3K222L, ScARO4K229L, and ScARO7T226I correspond to the feedback inhibited mutant of *S. cerevisiae* enzyme ScAro3, ScAro4, and ScAro7 [19].

*Yarrowia lipolytica* Strains Used in This Study			
Name	Genotype	Auxotrophy	Reference
JMY195, Po1d	*MATA ura3-302 leu2-270 xpr2-322*	Ura^−^, Leu^−^	[25]
JMY7997	Po1d + *LEU2ex-YlARO1-YlARO2* + *URA3ex-YlARO4K221L-YlARO7G139S* + *HPHex-ScARO3K222L* + *NATex-YlARO8-YlARO10*	Prototroph	This study
JMY8032	Po1d + *URA3ex-YlARO1-YlARO2* + *LEU2ex(recovered)ScARO4K229L-ScARO7T226I* + *LEU2ex-YlTKL*+ *NATex-YlARO8-YlARO10*	Prototroph	[19]
JMY8107	JMY8032 with *LEU2ex* and *NATex* marker recovered	Leu^−^	This study
JMY8131	JMY8107 Δ-*4hppd*	Prototroph	This study
JMY8178	JMY8032 + *HPHex-Yl4HPPD*	Prototroph	This study
JMY8208	JMY8032 + (*HPHex-Yl4HPPD*) multicopies	Prototroph	This study

## Data Availability

Raw sequencing data were deposited to the SRA database and are available under Project accession number: PRJNA715500.

## Supplementary Materials

The following are available online at https://www.mdpi.com/article/10.3390/microorganisms9040838/s1, Supplemental Figure S1: Acidification of the culture supernatant using HCl. After centrifugation, we compared how the pyomelanin pellet dissolved in water versus NaOH, Supplemental Figure S2: JMY8208 grown in YPD for 5 days and in YNB for 7 days, Supplemental Table S1: Oligonucleotides used in this study.
