# Supplementary material for: A Yarrowia lipolytica Strain Engineered for Pyomelanin Production
Source: Microorganisms. 2021 Apr 14;9(4):838. doi: 10.3390/microorganisms9040838 (PMC8071058; doi:10.3390/microorganisms9040838)
Supplement: Supplementary file 1 [file microorganisms-09-00838-s001.zip › Supplemental figure S1.pdf]

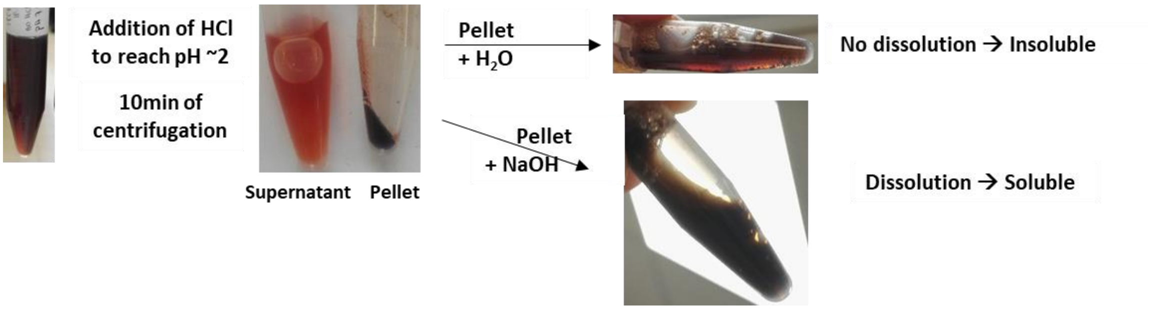

Supplemental Figure S1. Acidification of the culture supernatant using HCl. After centrifugation, we compared how the pyomelanin pellet dissolved in water versus NaOH.
