# Supplementary material for: A Yarrowia lipolytica Strain Engineered for Pyomelanin Production
Source: Microorganisms. 2021 Apr 14;9(4):838. doi: 10.3390/microorganisms9040838 (PMC8071058; doi:10.3390/microorganisms9040838)
Supplement: Supplementary file 1 [file microorganisms-09-00838-s001.zip › supplemental table S1.pdf]

Oligonucleotides		
Name	Sequence	
4HPPD_sgRNA1_Fw	TTCGATTCCGGGTCGGCGCAGGTTGGACCACGTCCACTGGTACGTGTTTTA	gRNA construction
4HPPD_sgRNA1_Rv	GCTCTAAAACACGTACCAGTGGACGTGGTCCAACCTGCGCCGACCCGGAAT	gRNA construction
GGP_G1_4HPPD_Fw	GGGGATCCGGTCTCTAATGTCACCTTCCGTCGAAGT	Gene amplification and adaptation to the GG system
GGP_G1_4HPPD_Rv	GGCCTAGGGGTCTCTTAGACTAAAGGTTGCCTCGCTTGG	Gene amplification and adaptation to the GG system
ForT7Verif_4HPPD_Fw	ATGTCACCTTCCGTCGAAGT	Verification of *4HPPD* disruption by Cas9
ForT7Verif_4HPPD_Rv	GAACTCGGTGCAAATGTCCT	Verification of *4HPPD* disruption by Cas9; verification of *4HPPD* cassette insertion into the genome
hph intern Fw	CATCCCTGAGGTTCTCGAC	Verification of *4HPPD* cassette insertion into the genome; verification of *scARO3K22L* cassette insertion into strain JMY7997
Aro1_insert3_Fw	CAACCTGGGCCTCAAGACCGCCGTTGAGC	Verification of *YlARO1-YlARO2* cassette insertion into strain JMY7997
Aro2_insert1_Rv	GCTCGCCGAGGCCGACGGGGCAGTTCCG	Verification of *YlARO1-YlARO2* cassette insertion into strain JMY7997
Aro4_insert4_Fw	TTGGGAGACTACCGTGGACATGCTCACCG	Verification of *YlARO4K221L-YlARO7G139S* cassette insertion into strain JMY7997
Aro7_G139S_insert1_Rv	ACCGAGGAATAGTTCTCGGGCTGATCTCCAG	Verification of *YlARO4K221L-YlARO7G139S* cassette insertion into strain JMY7997
Aro3scK222L_qRTPCR_Rv	CACTGAGCAGCCGTATTTCA	Verification of *scARO3K22L* cassette insertion into strain JMY7997
Aro8_qRTPCR_Fw	CAGATGGACGAGTTCAAGCA	Verification of *YlARO8-YlARO10* cassette insertion into strain JMY7997
Aro10_qRTPCR_Rv	CATCGTTGTTGAGCAGGAAA	Verification of *YlARO8-YlARO10* cassette insertion into strain JMY7997

Supplemental Table S1. Oligonucleotides used in this study.
