# Supplementary figures and images for: A Yarrowia lipolytica Strain Engineered for Pyomelanin Production
Source: Microorganisms. 2021 Apr 14;9(4):838. doi: 10.3390/microorganisms9040838 (PMC8071058; doi:10.3390/microorganisms9040838)

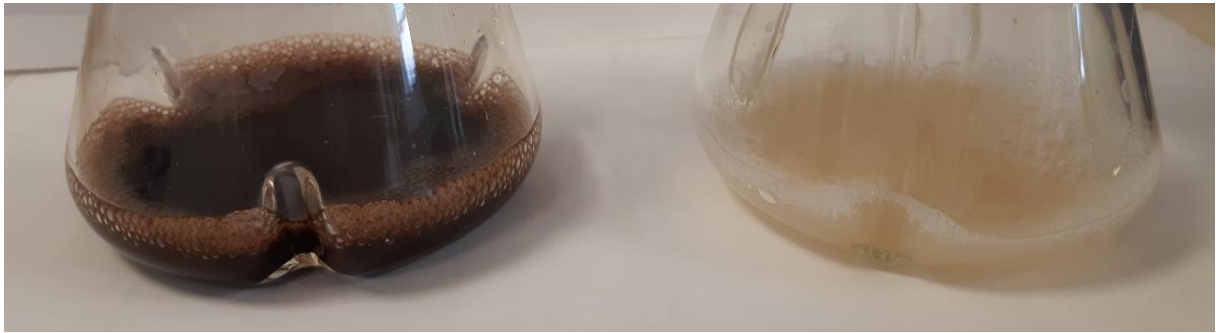

Supplemental Figure S2. JMY8208 grown in YPD for 5 days and in YNB for 7 days.

Supplement: Supplementary file 1 [file microorganisms-09-00838-s001.zip › Supplemental Figure S2.pdf]
